# Factors Affecting the Presence of Adequately Iodized Salt at Home in Wolaita, Southern Ethiopia: Community Based Study

**DOI:** 10.1155/2018/4394908

**Published:** 2018-03-22

**Authors:** Wondimagegn Paulos Kumma, Yusuf Haji, Junayde Abdurahmen, Yohannes Mehretie Adinew

**Affiliations:** ^1^College of Health Sciences and Medicine, Wolaita Sodo University, Sodo, Ethiopia; ^2^College of Medicine and Health Sciences, Hawassa University, Hawassa, Ethiopia; ^3^College of Medicine and Health Sciences, Madawalabu University, Goba, Ethiopia

## Abstract

**Background:**

Universal use of iodized salt is a simple and inexpensive method to prevent and eliminate iodine deficiency disorders like mental retardation. However, little is known about the level of adequately iodized salt consumption in the study area. Therefore, the study was aimed at assessing the proportion of households having adequately iodized salt and associated factors in Wolaita Sodo town and its peripheries, Southern Ethiopia.

**Methods:**

A cross-sectional study was conducted from May 10 to 20, 2016, in 441 households in Sodo town and its peripheries. Samples were selected using the systematic sampling technique. An iodometric titration method (AOAC, 2000) was used to analyze the iodine content of the salt samples. Data entry and analysis were done using Epi Info version 3.5.1 and SPSS version 16, respectively.

**Result:**

The female to male ratio of the respondents was 219. The mean age of the respondents was 30.2 (±7.3 SD). The proportion of households having adequately iodized salt was 37.7%, with 95% CI of 33.2% to 42.2%. Not exposing salt to sunlight with [OR: 3.75; 95% CI: 2.14, 6.57], higher monthly income [OR: 3.71; 95% CI: 1.97–7.01], and formal education of respondents with [OR: 1.75; 95% CI: 1.14, 2.70] were found associated with the presence of adequately iodized salt at home.

**Conclusion:**

This study revealed low levels of households having adequately iodized salt in Wolaita Sodo town and its peripheries. The evidence here shows that there is a need to increase the supply of adequately iodized salt to meet the goal for monitoring progress towards sustainable elimination of IDD.

## 1. Background

Iodine deficiency is one of the world's most important causes of preventable mental retardation [1]. It causes stunted growth and other developmental abnormalities, which are largely irreversible [2]. In later life, it reduces intellectual vigor, educational achievement, and productivity, which can be improved with increased iodine intakes [1, 2]. It has symptoms such as goiter and an abnormal enlargement of the thyroid gland [1]. There are almost no countries in the world where iodine deficiency was not a public health problem [2].

Goiter from iodine deficiency has been recognized for centuries and until recently it provided the primary means of assessing the extent of iodine deficiency disorders (IDD) [1, 2]. In 1994, United Nation Children's Fund (UNICEF) and World Health Organization (WHO) called on all countries to iodize salt, regardless of whether they had a documented IDD problem or not [1, 2]. The main driving force for change in the prevalence of iodine deficiency disorders is the coverage of iodized salt [2]. Globally, the proportion of people consuming iodized salt rose from less than 20 percent in 1990 to about 70 percent by 2000 [2]. Despite the fast global progress made in the proportion of people consuming adequately iodized salt (70%) by 2000, the figure remains stagnant since that date [2]. This lack of change since 2000 reveals the challenges that some countries are facing [1, 2]. According to an estimate made by UNICEF, every year in developing countries about 38 million newborns remain unprotected from the lifelong consequences of brain damage associated with iodine deficiency disorders [2]. This shortcoming affects a child's ability of learning and later life earning; thus, it prevents children, communities, and nations from fulfilling their dreams [2].

Goiter is the most prominent consequence of IDD. In Ethiopia, 62% of people are at risk of IDD from which goiter prevalence is estimated between 50% and 95% [3]. In south western Ethiopia, the gross goiter prevalence measured by palpation was 27.4% and the rate of cretinism and other milder disorders of IDD were estimated to be one and three out of 1000 persons, respectively [4]. Moreover, a study on risk factors of goiter in Sodo town, Southern Ethiopia, shows that 50.5% of the school children were suffering from goiter [5].

Universal salt iodization (USI) remains the key strategy to eliminate IDD. That means salt is used universally by all ages, socioeconomic, cultural, and religious groups throughout the year [1, 2]. Iodized salt is both a preventive and corrective measure for iodine deficiency and is the most effective, of low cost and long-term solution to a major public health problem [2]. Iodized salt should be used on a daily basis in an iodine deficient environment and the daily requirement of iodine for adults is 150 micrograms [2, 6].

In Ethiopia, though iodine is found naturally in top soil areas, in most areas of the country, especially in the highlands, the topsoil has been lost due to different factors such as deforestation, erosion, and flooding. And thus, food crops lack iodine content which in turn result in the dietary deficiency of iodine [3, 7]. According to Ethiopian Demographic and Health Survey, the proportion of households in Ethiopia using adequately iodized salt is only 15%; this indicates that the largest segment of the population is at risk of iodine deficiencies [3, 7]. This study considered demographic and health survey sites of the country in which our study area was not included.

A number of reasons are identified on why inadequately iodized salt is consumed at the household level. Rural residence, illiteracy, lower monthly per-capital income, poor socioeconomic status, unavailability of the salt, lack of awareness about the risk of IDD, using bulk (unpackaged) salt, exposing salt to sunlight, expensiveness, and lack of knowledge about iodized salt are among the factors associated with the absence of adequately iodized salt at home [7–14]. Therefore, the current study aimed at assessing the proportion of households that are having adequately iodized salt and the associated factors in Wolaita Sodo town and its peripheries in Southern Ethiopia.

## 2. Methods

### 2.1. Study Design and Setting

Community based cross-sectional study was conducted from March 10 to 20, 2016, in Wolaita Sodo town and its surrounding rural areas, Southern Ethiopia. Wolaita Sodo is located at 380 Km from the capital Addis Ababa. The study area is divided into 31 rural and 11 urban* kebeles *(the lowest administrative unit in Ethiopia). The total population of the study area in 2012/13 as projected based on the 2007 census data was 302,977 with the total household of 61,832 [15]. The source population was people residing in the selected administrative areas of Wolaita Sodo town and its surrounding rural areas.

### 2.2. Sample Size Determination and Sampling Techniques

A total of 441 households with adults aged 18 years and above were selected for the study using the assumptions of 15.4% proportion of iodinated salt at household level [7], 95% confidence level, 5% margin of error, design effect of 2, and 10% nonresponse rate. Systematic sampling technique was used to enroll households after 12 (9 rural and 3 towns)* kebeles* were randomly identified. The study* kebeles* and households were allocated proportionally for each stratum (town and rural residence). The member of the household who is responsible for purchasing food items and mostly involved in food preparation in the selected households was interviewed. In the absence of the selected households, revisit was arranged and the next house was considered if still absent.

### 2.3. Data Collection and Quality Control

Data were collected using interviewer administered structured questionnaire. The questionnaire was translated into local language and then back to English by different language experts to check for internal consistency. The tool contains sociodemographic section, environmental factors, and availability and accessibility of iodized salt. Six experienced diploma nurses collected the data under supervision of three BSc nurses. Two-day training was given both for the data collectors and supervisors. The tool was also pretested on adjacent* kebele* and necessary amendments were made.

### 2.4. Measurements

The iodine content of salt was determined by liberating iodine from salt and titrating the iodine with sodium thiosulphate using starch as an external indicator. An iodometric titration method (AOAC, 2016) [16] was used to analyze the iodine content of the salt samples in Ethiopian Public Health Institute Laboratory. Ten grams (10 g) of salt was dissolved in approximately 100 ml of distilled water. The pH was adjusted to 2.8 using 0.6% hydrochloric acid and 30 mg of potassium iodide powder was added to convert all the iodate present to free iodine. The liberated iodine was titrated with freshly prepared 0.004 M sodium thiosulphate solution using starch (freshly prepared) as the endpoint indicator. The thiosulphate level in the burette was recorded and iodine content in parts per million (ppm) was calculated using standard conversion table for iodine determination. The mean of the triplicate samples taken from each household was recorded [16].

### 2.5. Operational Definitions

According to WHO, adequately iodized salt for the sustainable elimination of IDD is defined as salt with iodine content of 15 parts per million (ppm) or more [17].

### 2.6. Data Entry, Analysis, and Processing

Data entry, cleaning, and coding were performed using Epi Info version 3.5.1 and then exported to SPSS version 16 for analysis. Descriptive statistics were done; moreover, bivariable and multivariable logistic regression analyses were also carried out. Associations between dependent and independent variables were considered and presented using odds ratios and 95% confidence intervals. Odds ratios and their 95% confidence intervals were computed and variables with *P* value < 0.05 were considered significant.

## 3. Results

### 3.1. Sociodemographic Characteristics of the Respondents

A total of 440 respondents were participated with a response rate of 99.8%. The study participants were from two districts, namely, Wolaita Sodo town administration (13.4%) and its surrounding rural district (86.6%). Nearly all (99.5%) of the respondents were women. About half (53.2%) of them were under the age category of 25–34 years. The mean age of the respondents was 30.2 (±7.3 SD) years. Most of the respondents (88%) were married (Table 1).

### 3.2. Household Practices of Respondents in Salt Utilization

Out of the total respondents, only 8 (1.8%) used salt for food preservation. Salting, drying, smoking, and pickling are some of food preservation methods used in Southern Ethiopia. About 391 (88.9%) of the participants reported that they were buying bulk (unpackaged) salt in general and 382 (86.8%) of the respondents reported that they were storing salt in dry places. Regarding the duration of salt storage at household level, the majority (77.7%) of them stored less than one week. Most (74.3%) of the respondents used a container with a cover, while few (13.4%) used a plastic bag to store salt at home. A high proportion (74.1%) of participants did not expose salt to sunlight and the majority (79.3%) of the households was buying salt every week. Concerning the time of adding salt to food, 26.1% added it at the beginning, 37.7% in the middle, and 36.1% at the end of cooking (Table 2).

Regarding the respondents' trend of buying iodized salt, only 138 (31.4%) of the respondents reported that they have a trend of buying iodized salt, of which 67 (48.6%) from local shops, 47 (34.1%) from open market, 14 (10.1%) from supermarket, and the remaining 10 (7.2%) from the zonal salt center. The zonal salt center is an enterprise recognized by the government as a store to sell salt for the locals on the market days. The reasons for buying iodized salt were health concern (2.5%), advice from health professionals (10.5%), and being the only salt in the area (0.5%). Out of the 302 (68.6%) participants who did not report as they have a trend of buying iodized salt, 140 (46.4%) said they did not have adequate information, 114 (37.8%) were not aware of the benefit, 42 (13.9%) of them said the salt was not found in their locality, and 37 (12.3%) of them said it is expensive, while few (6 (2%)) of them associated iodized salt with its taste related problem.

### 3.3. Result of Laboratory Test of the Salt

Out of the total salt samples, 180 (40.9%) of the samples had no iodine; about a quarter (108 (24.5%)) had 15–29.99 ppm; 79 (18%) had 7–14.99 ppm; 58 (13.2%) had 30 ppm or more and the remaining (3.4%) had less than 7 ppm. From the total salt samples, only 166 (37.7%; 95% CI: 33.2% to 42.2%) was adequately iodized, while the majority (62.3%) was inadequately iodized or not iodized at all (Table 3).

Among the 138 (31.4%) households who reported as buyers of iodized salt, 101 (73.2%) households were found to have salt of iodine content of 15 ppm or more. The remaining 26 (18.8%) and 11 (8.0%) households had salt of iodine content of 0 ppm and 1.00–14.99 ppm, respectively. Out of the 302 (68.6%) households who reported as not buyers of iodized salt, 65 (21.5%) households had salt of iodine content of 15 ppm or more. The remaining 154 (51.0%) and 83 (27.4%) households had salt of iodine content of 0 ppm and 1.00–14.99 ppm, respectively.

### 3.4. Factors Associated with the Presence of Adequately Iodized Salt

Tables 4 and 5 show factors associated with the presence of adequately iodized salt at home. Sociodemographic and other factors such as income level and practices were entered into binary logistic regression and then variables showing *P* value less than 0.2 were reentered into multivariable logistic regression to control for confounding. From the binary logistic regression analysis, place of residence with crude odds ratio (COR): 1.37 (1.02–1.83), formal education with COR: 2.22 (1.49–3.31), monthly income with *P* value less than 0.001, and not exposing salt to sunlight with COR: 2.7 (1.76–4.15) were positively associated with the presence of adequately iodized salt at home. However, variables such as family size, occupation, religion, age, type of salt bought, types of salt storage used, and salt storage places of the respondents were not associated with the presence of adequately iodized salt at home (Table 4).

On multivariable analysis, not exposing salt to sunlight and higher monthly income of respondents showed a strong relationship with the presence of adequately iodized salt at home. Participants who did not expose salt to sunlight were about four times [AOR: 3.75; 2.14–6.57] more likely to have adequately iodized salt as compared to their counterparts. Respondents' with monthly income of 60 United States Dollar (USD) (1201 Ethiopian Birr) or more were about four times [AOR: 3.71; 95 CI: 1.97–7.01] more likely to have adequately iodized salt as compared to low earners. Respondents' with formal education were about two times [AOR: 1.75; 1.14–2.70] more likely to have adequately iodized salt as compared to those who had no formal education (Table 5). However, in multivariable logistic regression, urban as place of residence with adjusted odds ratio (AOR): 0.88 (0.48–1.62) did not show association with the presence of adequately iodized salt at home (Table 5).

## 4. Discussion

Iodine deficiency disorders (IDD) are among the easiest and least expensive of all nutrient disorders to prevent [2, 7]. The addition of a small, constant amount of iodine to the salt that people consume daily is all that is needed. One of the goals for monitoring progress towards sustainable elimination of IDD as a public health problem determined by a Joint WHO/UNICEF/ICCIDD Working Group on assessment and monitoring of IDD is the percentage of households consuming adequately iodized salt that should be >90% [2, 16].

The current study included 440 households with the majority (391) from rural and the remaining 59 were from urban which is coincident with the Ethiopian rural/urban proportions. Out of the 440 households provided salt samples, adequately iodized salt was found only in 166 (37.7%) households using the titration method. The result of this study is higher than the findings observed in different parts of Ethiopia: Hawassa town 0% [18], Shebe town, south west Ethiopia 19% [19], Northern Ethiopia 25% [20], Gondar 29% [10], JigJiga town, Eastern Ethiopia 26.6% [21], and Laelay Maychew District, Northern Ethiopia 33% [22] of the households have access to iodized salt. The finding from the current study is also higher than the findings observed in Sudan 14.4% [9] and Sindh and Punjab provinces of Pakistan 15% [8]. The improvement observed in the current study might be attributed to the application of more effective strategies in the production and distribution of iodized salt to enhance universal salt iodization program by the Ethiopian Ministry of Health in general and zonal health department in particular.

However, this finding is lower than the findings from India 60.1% [23], Albania 60.41% [13], Ghana 75.6% [24], Kazakhstan 77.3% [25], and Nigeria 91% [26] of the households that were using adequately iodized salt. The explanation for the higher proportion of iodized salt consumption reported in these studies as compared to our finding might be because of the countries commitment towards increasing household accessibility to adequately iodized salt. The majority of the above findings is below the global target that requires more than 90% of the households to use adequately iodized salt [2, 17, 18]. This might be due to poor quality of the available salt or incorrectly iodized salt. The correctly iodized salt may also be deteriorating as a result of long-term exposure to sunlight, moisture, heat, contaminants, food processing, and washing and cooking processes in the household, which may result in losses of 50% or more of iodine content of the salt [2, 27]. The proportion of households having adequately iodized salt in this study site was low. This might be one of the reasons for the high prevalence of goiter (50.5%) observed in similar study site conducted by other researchers [5].

Respondents' practice of not exposing salt to sunlight showed an association with the presence of adequately iodized salt at home. Participants' who did not expose salt to sunlight were about four times more likely to have adequately iodized salt as compared to their counterparts. This is consistent with the findings of the study conducted in northern Ethiopia on the presence of adequately iodized salt at home and factors affecting it [10]. A study from India shows loss of 31% of iodine from iodized salt because of exposure to sunlight [28]. This might be due to the effect of heat on the iodine content. Or it might be due to the halogen nature of iodine and its exposure to excess oxygen and carbon dioxide slowly oxidizes it to metal carbonate and elemental iodine which then evaporates [29].

Respondents' monthly income is also associated with the presence of adequately iodized salt at home. Households with the monthly income of 60 USD or more were about four times more likely to have adequately iodized salt as compared to households earning lower. Similarly, Ethiopian Demographic and Health Survey shows that households in the highest wealth quintile were twice as likely to use iodized salt as compared to households in the lowest two wealth quintiles [7]. Similar survey in Sudan [9] shows that the wealthiest group of people consumes more iodized salt. And in India [11], households earning higher than 10 USD were more likely to use adequately iodized salt as compared to those who were earning lower. Moreover, the Albanian and Indian studies [13, 14] show association of poor socioeconomic status with the presence of inadequately iodized salt at home. This might be due to relatively high cost of adequately iodized salt to the poor.

Respondents' level of education is also associated with the presence of adequately iodized salt at home. Respondents with formal education were twice more likely to have adequately iodized salt as compared to respondents who had no formal education. This is consistent with other similar studies conducted on the households' use of iodized salt in Pakistan and India [8, 12]. This might be due to the fact that education improves access and use to iodized salt and this could be understood from the respondents' reasons for not buying iodized salt that majority cited lack of adequate information and awareness about its benefit.

Urban residence has shown association with the presence of adequately iodized salt at home in binary logistic regression; however, the association is lost after controlling for confounding. Other similar studies elsewhere reported the presence of an association between urban and the presence of adequately iodized salt at home [7–9, 11]. The reason may be that those who were unaware of its significance are the socially and economically disadvantaged rural population.

In the current study, 36% of mothers preparing food were adding iodized salt at the end of cooking. However, in order to serve its purpose, iodized salt must be stored in a dry place and added to a meal at the end of cooking [2, 6]. Regarding salt buying practices, the majority of households was buying salt every week because of the cost and weight of carrying and in most places there is only one market day a week. In the urban setting, it might be because of the availability of salt in their proximity and the possibility of buying in small quantity from retail shops. Out of the total households with the trend of buying iodized salt, 26.8% of the households had inadequately iodized salt. The explanation for this might be due to loss of iodine because of exposure to heat, sunlight, and moisture during storage at home or elsewhere in the supply chain [28, 29]. But also people may not get the appropriate product for the payment they make. In contrast, among the total households without the trend of buying iodized salt, 21.5% of the households had adequately iodized salt. Even though people may not buy adequately iodized salt intentionally, they may buy it because of its availability in their proximity and lower cost without having the knowledge that the salt they buy is adequately iodized.

## 5. Limitations of the Study

Being cross-sectional survey, it lacks temporal relationships. We did not test urinary iodine to see body iodine level. Testing salt only from households is not sufficient, as retail shops are the sources of iodized salt, where the households purchase and should be assessed to see whether the iodine content of the salt differs or not.

## 6. Conclusion

This study revealed low levels of households having adequately iodized salt in Wolaita Sodo town and its peripheries. Formal education, higher monthly income, and not exposing salt to sunlight were found to associate with the presence of adequately iodized salt at home. The evidence here shows that there is a need to increase the supply of adequately iodized salt to meet the goal for monitoring progress towards sustainable elimination of IDD.

## Figures and Tables

**Table 1:** Sociodemographic characteristics of the respondents, Wolaita Sodo town and its peripheries, Southern Ethiopia 2016.

Variables (*n* = 440)	Categories	Frequencies	Percentage
Place of residence	Urban	59	13.4
	Rural	381	86.6
Respondents' age	17–24 years	84	19.1
	25–34 years	234	53.2
	35–44 years	100	22.7
	45–54 years	20	4.5
	≥55 years	2	0.5
Sex of HH head	Female	47	10.7
	Male	393	89.3
Sex of respondents'	Female	438	99.5
	Male	2	0.5
Marital status	Married	397	88.0
	Widowed	31	7.0
	Single	13	3.0
	Divorced	9	2.0
Respondent's levels of education	Unable to read or write	200	45.9
	Only read and write	72	16.6
	Elementary school	101	22.0
	Secondary school	59	13.4
	College and above	8	2.0
Occupation	Housewife	319	72.5
	Private work	79	18.0
	Student	13	3.0
	Farmer	12	2.7
	Unemployed	9	2.0
	Government employee	8	1.8
Monthly income in ETB	≤600	221	57.0
	600–1200	126	28.6
	≥1201	63	14.3
Family size	1–3 persons	83	18.9
	4–6 persons	269	61.1
	>6 persons	88	20.0

HH: household; ETB: Ethiopian Birr.

**Table 2:** Households practices of respondents in salt utilization, Wolaita Sodo town and its peripheries, Southern Ethiopia 2016.

Variables (*n* = 440)		Frequencies	Percentage
Do you use iodized salt to preserve food?	Yes	8	1.8
	I do not preserve food	390	88.6
	I do not know what salt to use	42	9.6
What type of salt do you buy in general?	Bulk	391	88.9
	Packaged	49	11.1
Place of salt storage	Dry place	382	86.8
	Moisture area	36	8.2
	Fire area	22	5.0
Duration of salt storage at household level	<1 week	342	77.7
	1–4 weeks	89	20.2
	5–9 weeks	7	1.6
	>10 weeks	2	0.5
What type of salt storage do you use?	Storage with cover	327	74.3
	Storage without cover	52	11.8
	Plastic salt bag	59	13.4
	Other s	2	0.5
Do you expose salt to sun light?	Yes	114	25.9
	No	326	74.1
How often do you buy salt?	Once in a week	349	79.3
	Once a month	91	20.7
When do you add salt to food?	At the beginning	115	26.1
	In between cooking	166	37.7
	At the end of cooking	159	36.1

**Table 3:** Chemistry of salt samples from households in Wolaita Sodo town and its peripheries, Southern Ethiopia 2016.

Variables (*n* = 440)	Categories	Frequencies	Percentage
Iodometric test range	0 ppm	180	40.9
	<7 ppm	15	3.4
	7–14.99 ppm	79	18.0
	15–29.99 ppm	108	24.5
	>30 ppm	58	13.2
	Adequately iodized (≥15 ppm)	166	37.7
	Inadequately iodized/no iodine (<15 ppm)	274	62.3

ppm: parts per million.

**Table 4:** Sociodemographic and practices factors associated with the presence of adequately iodized salt at home in binary logistic regression, Wolaita Sodo town and its peripheries, Southern Ethiopia 2016.

Variables (*n* = 440)	Titration result of the salt		COR	*P* value
	≥15 ppm	<15 ppm		
	No (%)	No (%)		
Place of residence				
Urban	29 (17.5)	30 (11)	1.37 (1.02–1.83)	
Rural	137 (82.5)	244 (89)	1	0.052
Educational status				
No formal education	83 (50.0)	189 (69.0)	1	
Formal education	83 (50.0)	85 (31.0)	2.22 (1.49–3.31)	<0.001
Family size				
≤5	100 (60.2)	158 (57.7)	1	
>5	66 (39.8)	116 (42.3)	1.07 (0.84–1.38)	0.59
Religion of respondents				
Protestant	103 (62.0)	160 (58.4)	1	
Orthodox	54 (32.5)	94 (34.3)	1.43 (0.63–3.26)	0.64
Others	9 (5.4)	20 (7.3)	1.28 (0.54–3.00)	
Occupation				
Housewife	120 (72.3)	199 (72.6)	0.72 (0.30–1.72)	
Employed	36 (21.7)	63 (23.0)	0.69 (0.27–1.74)	
Unemployed	10 (6.0)	12 (4.4)	1	0.72
Age of respondents				
<25 years	25 (15.1)	59 (21.5)	0.61 (0.23–1.61)	
25–34 years	94 (56.6)	140 (51.1)	0.97 (0.41–2.36)	
35–44 years	38 (22.9)	62 (22.6)	0.88 (0.34–2.27)	0.39
≥45 years	9 (5.4)	13 (4.7)	1	
Income of the respondents				
≤600 ETB	72 (43.8)	179 (65.3)	1	
601 = 1200 ETB	52 (31.3)	74 (27.0)	1.75 (1.12–2.73)	
≥1201 ETB	42 (25.3)	21 (7.7)	4.97 (2.75–8.98)	<0.001
Type of salt bought				
Packaged	20 (41)	29 (59)	0.86 (0.47–1.58)	0.63
Bulk	146 (37)	245 (63)	1.00	
Types of salt storage used				
Storage with cover/salt bag	149 (39)	237 (61)	0.73 (0.4–1.34)	0.31
Storage without cover	17 (31)	37 (68.5)	1.00	
Exposed salt to sun light				
Yes	19 (17)	95 (83)	1.00	<0.001
No	147 (45)	179 (55)	2.7 (1.76–4.15)	
How often do you buy salt				
Once in a month	33 (36)	58 (64)		
Once in a week	133 (38)	216 (62)	1.08 (0.67–1.75)	0.75
Place of salt storage				
Dry place	141 (85)	241 (88)	1.3 (0.74–2.27)	0.36
Moisture/fire area	25 (15)	33 (12)	1.00	

ppm: parts per million, COR: crude odds ratio, AOR: adjusted odds ratio, and ETB: Ethiopian Birr.

**Table 5:** Sociodemographic and practices factors associated with the presence of adequately iodized salt at home in multivariable logistic regression, Wolaita Sodo town and its peripheries, Southern Ethiopia 2016.

Variables (*n* = 440)	Salt titration result		COR	AOR
	≥15 PPM	<15 PPM		
	No (%)	No (%)		
Place of residence				
Urban	29 (17.5)	30 (10.9)	1.37 (1.02–1.83)	0.88 (0.48–1.62)
Rural	137 (82.5)	244 (89.1)	1	1
Educational status				
No formal education	83 (50.0)	189 (69.0)	1	1
Formal education	83 (50.0)	85 (31.0)	2.22 (1.49–3.31)	1.75 (1.14–2.70)
Income of the respondents				
≤600 ETB	72 (43.8)	179 (65.3)	1	1
601 = 1200 ETB	52 (31.3)	74 (27.0)	1.75 (1.12–2.73)	1.35 (0.84–2.16)
≥1201–1800 ETB	42 (25.3)	21 (7.7)	4.97 (2.75–8.98)	3.71 (1.97–7.01)
Exposed salt to sun light				
Yes	19 (17)	95 (83)	1.00	1.00
No	147 (45)	179 (55)	2.7 (1.76–4.15)	3.75 (2.14–6.57)

ppm: parts per million, COR: crude odds ratio, AOR: adjusted odds ratio, and ETB: Ethiopian Birr.

## Data Availability

The data set used for the current article is available from the corresponding author on reasonable request.

## Abbreviations

ICCIDD: International Council for Control of Iodine Deficiency Disorders
IDD: Iodine deficiency disorders
WHO: World Health Organization
UNICEF: United Nation Children's Fund
USI: Universal salt iodization
USD: United States Dollar.

## References

[1] United Nation System Standing Committee on Nutrition (2009). *Progress in Nutrition: Sixth Report on the World Nutrition Situation*.

[2] UNICEF (2008). *Sustainable Elimination of Iodine Deficiency: Progress Since the 1990 World Summit for Children*.

[3] FMOH (2010). *National Guideline for Control and Prevention of Micronutrient Deficiencies*.

[4] Berhanu N., Michael K. W., Bezabih M. (2005). Endemic goiter in school children in Southwestern Ethiopia. *Ethiopian Journal of Health Development*.

[5] Wolka E., Shiferaw S., Biadgilign S. (2014). Epidemiological study of risk factors for goiter among primary schoolchildren in southern Ethiopia. *Food and Nutrition Bulletin*.

[6] WHO (2001). *Assessment of Iodine Deficiency Disorders and Monitoring Their Elimination: A Guide for Programme Managers*.

[7] Central Statistical Agency (CSA) (2012). *Ethiopia Demographic and Health Survey (EDHS), 2011*.

[8] Khan G. N., Hussain I., Soofi S. B., Rizvi A., Bhutta Z. A. (2012). A study on the household use of iodised salt in sindh and punjab provinces, Pakistan: Implications for policy makers. *Journal of Pharmacy and Nutrition Sciences*.

[9] Mahfouz M. S., Gaffar A. M., Bani I. A. (2012). Iodized salt consumption in Sudan: present status and future directions. *Journal of Health, Population and Nutrition*.

[10] Gebremariam H. G., Yesuf M. E., Koye D. N. (2013). Availability of adequately iodized salt at household level and associated factors in Gondar Town, Northwest Ethiopia. *ISRN Public Health*.

[11] Sen T. K., Das D. K., Biswas A. B., Chakrabarty I., Mukhopadhyay S., Roy R. (2010). Limited access to iodized salt among the poor and disadvantaged in North 24 Parganas district of West Bengal, India. *Journal of Health, Population and Nutrition*.

[12] Abedi A. J., Srivastava J. P. (2013). Consumption of iodized salt among households of District Lucknow, India. *International Journal of Advanced Research*.

[13] Kadiu E., Guri N., Hyskaj D. R. J. (2014). Determination of concentration of the iodine in household salt in Albania. *American Journal of Engineering Research*.

[14] Panigrahi A., Mishra K., Mohapatra B. (2009). Status of iodized salt coverage in urban slums of Cuttack City, Orissa. *Indian Journal of Community Medicine*.

[15] Population Census Commissions (2008). *Summary and Statistical Report of the 2007 Population and Housing Census: Population Size by Age and Sex*.

[16] AOAC Official Methods of Analysis, Appendix F: guidelines for standard method performance requirements, 2016, http://www.eoma.aoac.org/app_f.pdf

[17] WHO (2001). *Assessment of Iodine deficiency Disorders and Monitoring their Elimination: A Guide for Programme Managers*.

[18] Girma M., Loha E., Bogale A., Teyikie N., Abuye C., Stoecker B. J. (2012). Iodine deficiency in primary school children and knowledge of iodine deficiency and iodized salt among caretakers in Hawassa Town: Southern Ethiopia. *Ethiopian Journal of Health Development*.

[19] Takele L., Belachew T., Bekele T. (2003). Iodine concentration in salt at household and retail shop levels in Shebe town, South West Ethiopia. *East African Medical Journal*.

[20] Shawel D., Hagos S., Lachat C. K., Kimanya M. E., Kolsteren P. (2010). Post-production losses in iodine concentration of salt hamper the control of iodine deficiency disorders: a case study in Northern Ethiopia. *Journal of Health, Population and Nutrition*.

[21] Tahir A., Seyoum B., Kadir H. (2016). Use of iodized salt at household level in Jig Jiga Town, Eastern Ethiopia. *Asian Journal of Agriculture and Life Sciences*.

[22] Gidey B., Alemu K., Atnafu A., Kifle M., Tefera Y., Sharma H. R. (2015). Availability of adequate iodized salt at household level and associated factors in rural communities in Laelay Maychew District, Northern Ethiopia: a cross sectional study. *Journal of Nutrition and Health Sciences*.

[23] Patro B., Saboth P., Zodpey S., Shukla A., Karmarkar M. G., Pandav C. S. (2008). Tracking progress toward elimination of iodine deficiency disorders in Jharkhand, India. *Indian Journal of Community Medicine*.

[24] Buxton C., Baguune B. (2012). Knowledge and practices of people in Bia District, Ghana, with regard to iodine deficiency disorders and intake of iodized salt. *Archives of Public Health*.

[25] UNICEF (2005). *An Assessment of the Household Use and Adequacy of Iodized Salt in the Republic of Kazakhsta*.

[26] Kunle A., Olanrewaju S. A. (2014). Evaluation of the iodine content of table salt in Ado-Ekiti, Nigeria. *International Journal of Novel Research in Engineering and Applied Sciences*.

[27] World Health Organization. Guiding for a National program for the control of Iodine. Jeneva Switzerland: World Health Organization; 1994

[28] Kapil U., Prakash S., Nayar D. (1998). Study of some factors influencing losses of iodine from iodised salt. *Indian Journal of Maternal and Child Health*.

[29] Waszkowiak K., Szymandera K. (2008). Effect of storage conditions on potassium iodide stability in iodised table salt and collagen preparations. *International Journal of Food Science & Technology*.

